# High iodine content in local animal milk and risk of exceeding EFSA upper intake level for iodine among Saharawi women

**DOI:** 10.1371/journal.pone.0212465

**Published:** 2019-02-15

**Authors:** Marianne S. Morseth, Inger Aakre, Ingrid Barikmo, Lisbeth Dahl, Sigrun Henjum

**Affiliations:** 1 Department of Nursing and Health Promotion, Faculty of Health Sciences, OsloMet – Oslo Metropolitan University, Oslo, Norway; 2 Institute of Marine Research (IMR), Bergen, Norway; Università degli Studi di Milano, ITALY

## Abstract

Excessive iodine intake is a major public health problem in the Saharawi refugee camps in Algeria, where animal milk is an important source of iodine. The purpose of this study was to assess the association between iodine concentrations in locally produced animal milk and in animal drinking water and further to assess the risk of exceeding European Food Safety Authority (EFSA) tolerable upper intake level for iodine (600 μg/d) among Saharawi women. In 2009 and 2010, 202 milk samples from goats and sheep and 52 milk samples from camel were collected. Iodine in milk was determined by Inductively Coupled Plasma-Mass Spectrometry (ICP-MS). In addition, iodine in 56 water samples from the general water system and 54 water samples from wells, was determined by modified Sandell-Kolthoff reaction. Animal milk is generally consumed mixed with water. The median (min, max) iodine intake from goat/sheep milk mixed with water in camps with high iodine content in human drinking water was 284 (57, 2889) μg/d and 19% of participants exceeded EFSA upper intake level for iodine. The median (min, max) iodine intake from camel milk mixed with water in all camps was 2100 (210, 11100) and 47% of participants exceeded the EFSA upper intake level. The median (min, max) iodine content in goat/sheep milk from camps with moderate and high iodine content in water was 507 (101, 4791) μg/L and 1612 (487, 9323) μg/L, respectively (p < 0.001). The iodine content in goat/sheep milk was positively associated with iodine content in animal drinking water (regression coefficient, B 5.71, 95% CI 4.03, 7.39). In conclusion, consumption of camel milk and living in camps with high water iodine content increased the risk of exceeding the EFSA upper intake level for iodine. We suggest that purified water for both human and animal consumption should be provided.

## Introduction

The Saharawi refugees are suffering from one of the world’s “forgotten” international conflicts. Since 1975, approximately 165 000 people [1] have been living in refugee camps in a harsh desert climate, near Tindouf, Algeria. One main concern in this protracted situation has been water supply and quality. Initially, shallow groundwater wells were used, but due to strong rains in 1994, these were contaminated, and the use of deeper groundwater wells was initiated. Since the year 2000, raw water treatment by reverse osmosis has been used which reduces iodine levels in the water [2].

Both low and high intakes of iodine are associated with thyroid dysfunction [3]. While there has been a major decline in iodine deficient countries over the past two decades, the number of countries with excessive iodine status has increased in the last 10 years. In 2017, 11 countries reported iodine excess [4]. Milk and dairy products are important sources of iodine in Western countries, but the iodine content of milk varies widely depending on i.e the iodine content of animal feed, goitrogen intake, milk yield and season [5, 6]. Other sources of iodine are nutritional supplements, water, seaweed, medications and iodized salt [7, 8]. Safe upper intake level for iodine (600 μg/d by European Food Safety Authority (EFSA) and 1100 μg/d by the United States Institute of Medicine [9] is defined as the highest level of daily consumption that is considered safe for, and cause no side effects in, 97.5% of healthy adults [9, 10]. Although the thyroid has many mechanisms to adapt to excessive iodine intakes, hypothyroidism may result from excessive intakes in vulnerable groups who lack these adaptive mechanisms, such as individuals with previous thyroid disease or thyroid autoimmunity [11] or developing foetuses [7]. Iodine excess may also cause transient or permanent hyperthyroidism in individuals with previous thyroid disease or iodine deficiency [11]. The hyperthyroidism likely stems from increased lymphoid infiltrates in the thyroid, similar to changes seen in iodine deficiency [12, 13]. Animal models suggest that iodine excess is associated with thyroid autoimmunity [8], and both low and high iodine intakes are associated with risk of thyroid cancer in both humans and animals [14]. Finally, thyroid hormones influence brain development through myelinization, migration, maturation and differentiation of nerve cells [15]. Even though both iodine deficiency and iodine excess are known to affect thyroid hormone synthesis similarly, very little research has been dedicated to excessive iodine intakes and child development. However, a series of Chinese studies show mixed results for iodine excess and intelligence scores in children [16–19].

Excessive iodine intake is a major public health problem in the Saharawi refugee camps, and has been associated with thyroid dysfunction [20–22] and poor developmental status in children [23] in this population. A study from 2007 showed that main sources of iodine among Saharawi women was water and locally produced animal milk [24]. While the iodine content in water has been reduced over the past years, goitre prevalence has remained high and a call for research into other dietary sources such as animal milk, has been voiced [25]. The majority of Saharawi households own livestock, and goats and sheep [26] are most common. In a survey from 2010, about 60% of mothers had consumed local animal milk in the past 24 hours [27], which indicates that it is an important part of Saharawi food culture. High iodine content in animal milk has previously been reported in this area [24]. However, the reasons for high iodine content in animal milk and risk of too high iodine intake from animal milk have not been adequately assessed in the Saharawi refugee camps. The purpose of this study was therefore to assess the association between iodine concentrations in locally produced animal milk, animal drinking water and further to assess the risk of exceeding European Food Safety Authority (EFSA) tolerable upper level for iodine intake (600 μg/d) among Saharawi women.

## Material and methods

### Subjects

The data in this paper originates from two cross-sectional studies conducted in 2009 and 2010 (hereafter referred to as study 1 and study 2), among Saharawi refugees in four camps (El Aiun, Awserd, Smara/Boujdour and Dakhla) in the area around Tindouf, Algeria and the Free zone close to the Mauritanian border. In the camps, animal milk is for the most part consumed mixed with water, while in the Free Zone milk is more commonly consumed pure. Currently, El Aiun and Awserd receive 20 days of purified water and 20 days of ground well water (not purified) in cycles, Smara and Boujador receive purified water continuously, while Dakhla receive water from local wells with naturally lower level of iodine [2]. In the Free zone, only wells are used for drinking water. In study 1, the aim of the data collection was to acquire samples of animal milk (goat/sheep and camel), drinking water used by humans and animals and iodine intake data from milk mixed with water among Saharawi women. We targeted households who owned livestock. Within these households, milk intake was primarily assessed among mothers/caregivers of children <12 years old, or the youngest woman > 16 years in household with no children. Study 2 was included to increase the number of camel milk samples. The study was part of a larger nutrition and iodine survey among breastfeeding women with children 0–6 months. For further description, see Aakre et al. [21].

### Sample selection

According to the livestock census from 2007, there were approximately 62.000 goats and sheep in the camps. Based on a 95% confidence level and confidence interval of seven, the sample size was calculated to be 200 animals from the 5 camps and 20 from the Free Zone. Households were included based on random sampling. The number of households to count between each included household was based on the proportion between the number of families receiving the basic food ration (a proxy for total number of families) and the estimated number of families owning goats or sheep (livestock census) in the same camp. About half the families first approached were unable to provide a milk sample. In these cases, we asked if they knew of any neighbouring households that were eligible and included these instead. From families who owned both goats/sheep and camels, samples from both species were collected. Since camels were scarce in most camps, we also asked the participants/families whether they had neighbours with camels and then included these. Finally, due to long distances in the Free Zone and limited time for data collection, households from this area were selected by convenient sampling. The included households were from Tifariti, Bir-lehlu and Bir-tiguisit.

### Milk and water samples

The milk was collected from plastic bottles used by families for storage in pre-labelled 50 mL plastic containers immediately after the interview if the family had milk ready or was picked up the next day. Milk samples were kept frozen pending analysis. Water samples from the general water system (n = 56) and from wells (n = 54) were collected in 2009 and 2010 and kept in 50 mL plastic containers pending analysis.

### Laboratory analysis

The determination of iodine in the water samples was carried out at the Iodine Nutrition Laboratories, Nutritional Intervention Research Unit, and South African Medical Research Council in Cape Town, South Africa. A slightly modified quantitative method based on the Sandell-Kolthoff reaction [28] was used. The water samples were first digested with ammonium persulfate [29] to remove interfering substances and then incorporated to a microplate application for the spectrophotometrically determination of the inverse colour reaction at 405 nm on a micro plate reader [30]. The laboratory participates three times/year in an external quality program EQUIP [31]. Iodine concentration in milk was determined in duplicates by Inductively Coupled Plasma-Mass Spectrometry (ICP-MS) after digestion in tetra methyl ammonium hydroxide at the Institute of Marine Research (IMR) (previously National Institute of Nutrition and Seafood Research (NIFES)) in Norway. Limit of quantification is 0.32 μg/L. The accuracy of the results was verified with certified reference material selected with regard to similarity in concentration and matrix to sample material analysed and is further described elsewhere [32, 33].

### Milk intake

Milk intake was assessed using pre-coded questionnaires (S1 and S2 Questionnaires)[27]. In study 1, for goat/sheep- and camel milk separately, frequency of consumption of animal milk mixed with water in the past 24 hours, amount of milk and water used in the mix and amount of mix consumed was assessed. Amounts were estimated using measuring cups (1 litre) and plastic bottles (1.5 litres). Iodine content analysed in milk and human drinking water (μg/L) was used to calculate total iodine intake (μg /d) and risk of exceeding upper intake levels.

### Animal feed

Animal feed was only assessed in study 1 using a questionnaire with preformed alternatives: household food waste, carton, garbage, fresh greens and dried greens. More than one alternative could be ticked off (S3 and S4 Questionnaires).

### Statistics

Data was analysed using Statistical Package for Social Sciences (SPSS) version 24.0. Since all variables were highly skewed, with some extremely high values, median (min, max) values are reported. Differences between iodine content in milk from areas with moderate- and high iodine content in water was assessed by Mann-Whitney U-test. The association between iodine in water and iodine in animal milk was assessed using linear regression analysis. In assessing the risk of excessive iodine intake, areas with a continuous supply of purified water (Smara/Boujador) and naturally lower iodine content in water (Dakhla) were defined as moderate water iodine areas, while areas receiving a mix of purified and unpurified water (El Aiun and Awserd) were defined as high water iodine areas. Due to few samples of camel milk in the 2009 study, regression analysis on iodine in water and iodine in camel milk was not performed. Also, due to uncertainty about the origin of camel milk consumed in study 2 [21], analyses for camel milk was performed for all camps combined.

### Ethics

Ethics approval for the survey was given by the Regional Committees for Medical and Health Research Ethics in Norway (ref. 2010/2513) and by the Saharawi Ministry of Public Health. Informed consent was obtained both orally and in writing from all participants. It was emphasized that refusal to participate or withdrawal from the survey would not have any negative consequences for the participants.

## Results

Iodine content in water and milk samples is presented in Table 1. The median (min, max) iodine content in goat/sheep milk from areas with moderate and high iodine content in water was 507 (101, 4791) μg/L and 1612 (487, 9323) μg/L (p < 0.001), respectively. The median (min, max) iodine content in camel milk was 2107 (210, 11100) μg/L.

**Table 1:** Iodine content in water and animal milk.

	Water				Goat/sheep milk^a^		Camel milk^b^	
	General system^b^		Wells^a^^,^^c^		N	Iodine (μg/L), median (min, max)	N	Iodine (μg/L), median (min, max)
	Iodine (μg/L)		Iodine (μg/L)					
	N	Median (min, max)	N	Median (min, max)				
El Aiun	10	267 (94, 376)	33	273 (46, 986)	56	1980 (589, 9323)	16	1952 (664, 6574)
Awserd	9	240 (118, 280)	0	NA	43	1138 (487, 5485)	10	1810 (1150, 11100)
Smara	14	44 (36, 105)	0	NA	69	468 (101, 3170)	17	2930 (357, 6470)
Boujador	14	44 (36, 105)^d^	0	NA	7	1727 (427, 4791)	1	2490
Dakhla	9	80 (44, 83)	1	242	13	362 (247, 1268)	3	525 (210, 1664)
Free zone	0	NA	20	122 (25, 730)	14	2326 (1221, 3112)	5	5150 (3823, 7799)
Moderate water iodine area^e^	23	253 (94, 376)			89	507 (101, 4791)		NA
High water iodine area^f^	19	74 (36, 105)			99	1612 (487, 9323)		NA
Total		NA	53	210 (25, 986)	202	1015 (101, 9323)	52	2107 (210, 11100)

Saharawi refugee camps, Tindouf, Algeria, 2009

^a^ Only samples from 2009

^b^ Samples from 2009 and 2010 combined, milk samples from 2010 was from own livestock or purchased

^c^ Wells providing water to animals

^d^ Same water system as in Smara

^e^ Smara, Boujador and Dakhla

^f^ El Aiun and Awserd

The iodine content of water used for animals was significantly associated with iodine content in goat/sheep milk (B 5.71, 95% CI 4.03, 7.39), explaining 18% of the variation of iodine content in the goat/sheep milk. Iodine in water was negatively correlated with iodine in camel milk, Spearman regression coefficient -0.46 (p = 0.06).

**Fig 1:**
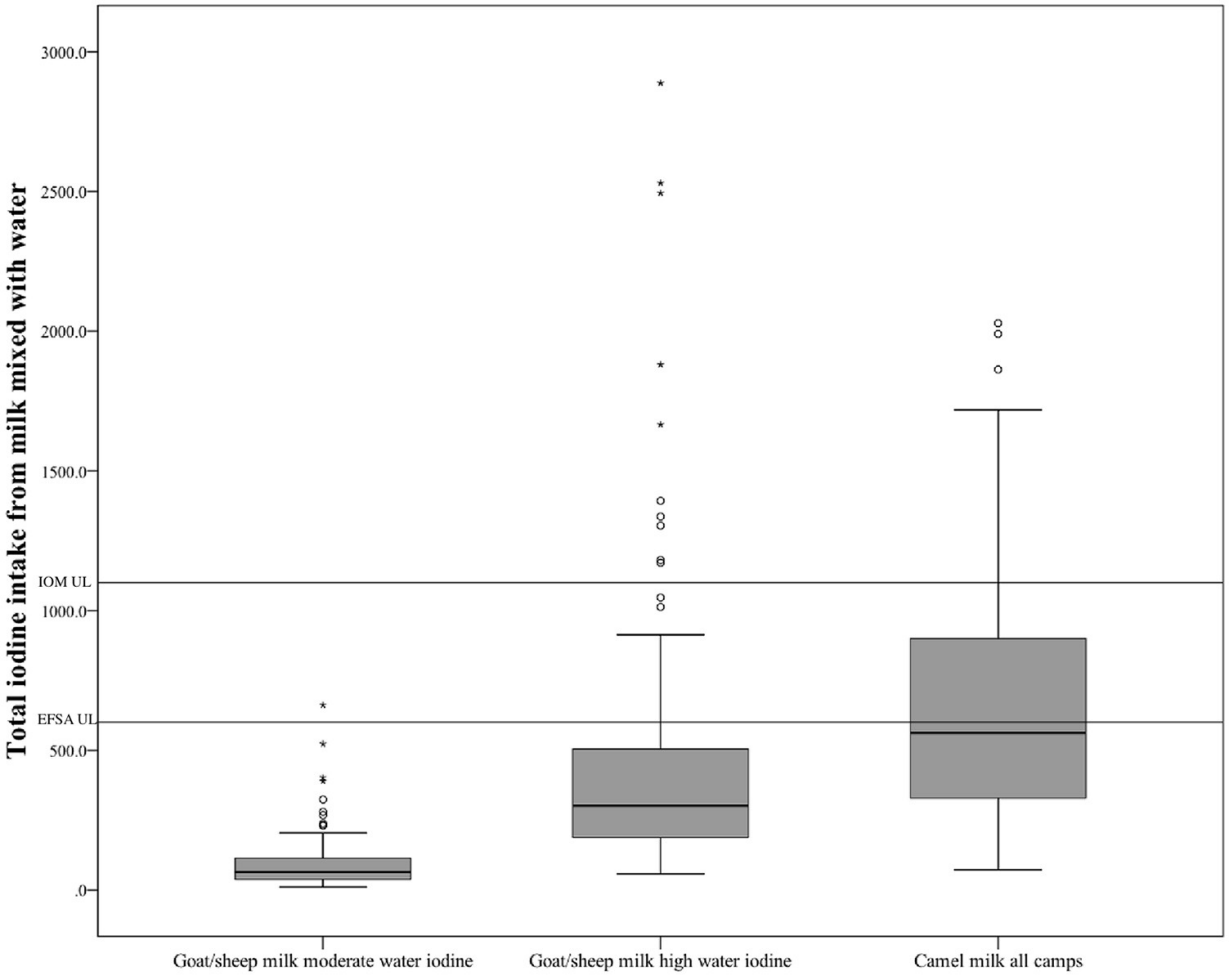
Iodine intake. Box plots for daily iodine intake (μg/d) from goat/sheep (n = 89 in moderate and n = 97 in high water iodine areas) and camel (n = 47 samples, all camps minus the Free Zone) milk mixed with water among women, Saharawi refugee camps, 2009–2010.

Fig 1 shows iodine intakes from goat/sheep milk mixed with water among women in areas with moderate and high iodine content in water and iodine intakes from camel milk mixed with water from all camps (minus the Free Zone). The median (min, max) iodine intake from goat/sheep milk mixed with water in areas with moderate and high- water iodine content was 60 (10, 523) μg/d, and 284 (57, 2889) μg/d, respectively. The median (min, max) iodine intake from camel milk mixed with water was 588 (72, 2029).

No women were at risk of excessive intake from goat/sheep milk mixed with water in areas with moderate water iodine content. Nineteen percent were at risk of exceeding the EFSA upper intake level (600 μg/d) and 8% the IOM upper intake level (1100 μg/d) from goat/sheep milk mixed with water in areas with high water iodine content. Corresponding numbers for camel milk (all camps) was 47 and 21%, respectively. The median (min, max) iodine intake in the group of women exceeding the EFSA upper intake level (n = 44) from milk (any type) mixed with water was 982 (606, 2889) μg/d.

**Fig 2:**
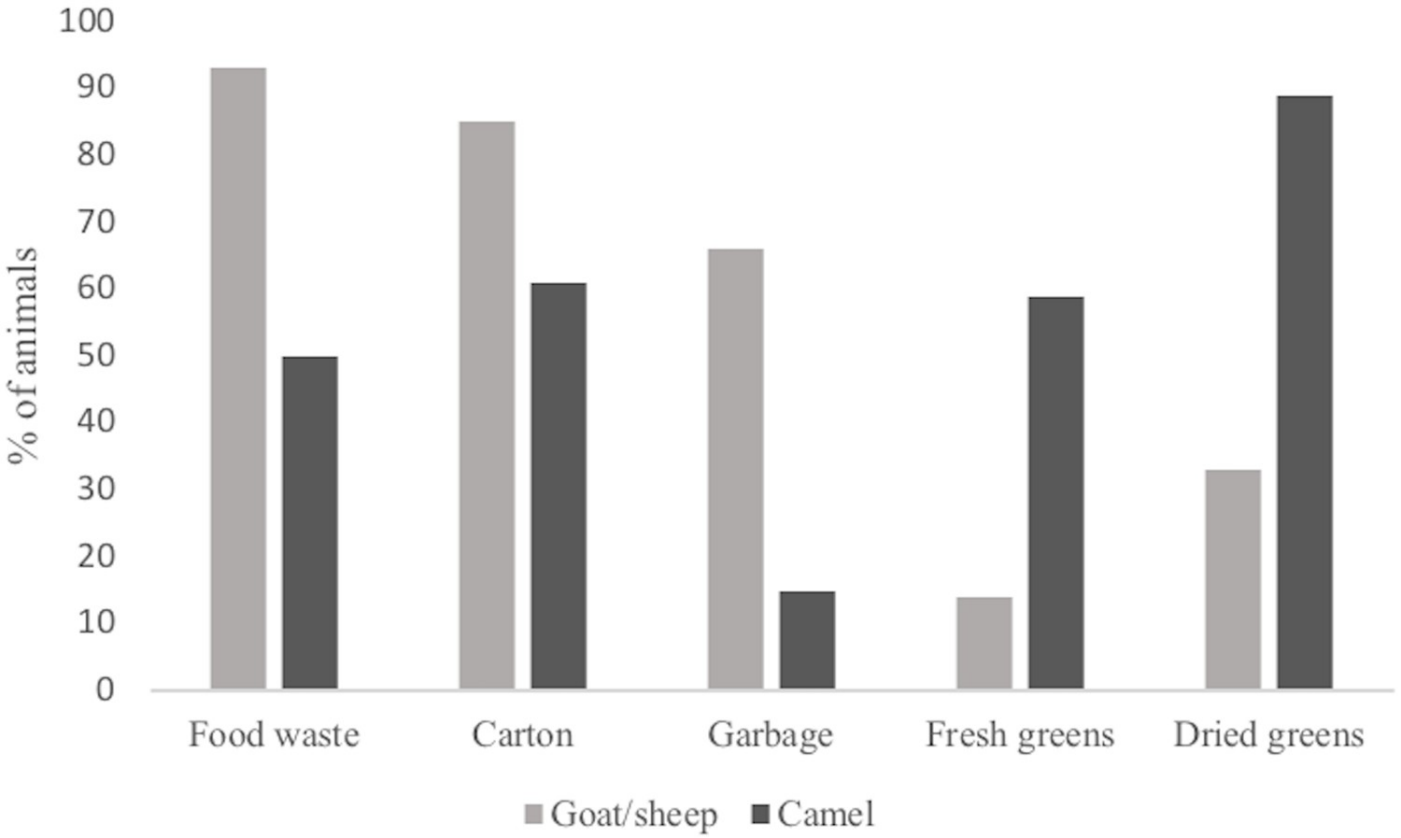
Animal feed. Type of feed consumed by goats/sheep (n = 202) and camels (n = 18) in Saharawi refugee camps and the Free zone, November 2009.

Type of feed consumed by goats/sheep and camels is presented in Fig 2. While almost all (80–90%) goats/sheep ate household food waste and carton, for camels, the majority had eaten fresh greens and almost all (90%) had eaten dried greens.

## Discussion

Consumption of camel milk and consuming goat/sheep milk from an area with high water iodine content greatly increased the risk of exceeding EFSAs upper intake level for iodine among women. The iodine concentration in camel milk was in general higher than in goat/sheep milk. Iodine concentration in goat/sheep milk was positively associated with water iodine.

### Risk of exceeding upper intake level of iodine

The window for optimal intake of iodine is narrow [3], and excessive iodine intake may have harmful effects on human health [7, 34, 35]. In this study, the risk of consuming more than the upper intake levels for iodine from animal milk mixed with water was highest after consumption of camel milk and consuming goat/sheep milk from areas with high iodine content in water. Camel milk is frequently sold from small- and large scale commercial producers, many living in the Free Zone [36]. Based on our study, consumption of 1–2 dL of this milk daily (if consumed pure) will be enough to exceed the EFSA upper intake level of iodine. The high prevalence of women exceeding the EFSA upper intake level from camel milk mixed with water is further disturbing given that animal milk is not the only source of iodine in the Saharawi diet [27]. The fact that milk with extremely high iodine levels is consumed by so many in the camps is problematic and must be further addressed in co-operation with Saharawi authorities. On the one hand, the nutrient content of animal milk is beneficial [37], and camel milk in particular is highly valued due to Saharawi heritage and belief in health-promoting properties [36]. On the other hand, this must be balanced against the risk of iodine overconsumption.

### Iodine in milk

Camel milk had in general higher iodine content than goat/sheep milk, even when the same water source was used. This could possibly be caused by differences in iodine content in feed used for camels and goats. For instance, locally produced fresh greens irrigated with iodine-rich water, commonly fed to camels, would likely have a high iodine content [38] compared to food waste and carton more commonly consumed by goats/sheep. Further, the iodine content in animal drinking water was associated with iodine content in goat/sheep milk, but not in camel milk. This may be due to few samples of camel milk or different physiology in the species assessed. Camels inhabiting desert climates (Camelis dromedarus) are more adapted to conserving water than other types of herd [39]. Iodine is primarily excreted through urine and through milk when animals are lactating [40]. In order to sustain the nutrient needs of their offspring, camels will, in times of water scarcity, produce milk with increased levels of salts [41]. From an evolutionary point of view, it seems likely that this also applies to other minerals including iodine. Further, similar to humans [42], animals may lack regulatory feedback mechanisms in milk glands, which may lead to a concentrated iodine level in milk when exposed to high levels of dietary iodine. Apart from iodine intake from feed, the iodine content in cow’s milk is also associated with lactation stage and milk yield [6]. No previous studies, to our knowledge, have investigated how the iodine content of camel milk might change in relation to lactation period or hydration status. In this setting with high iodine content in some animal drinking water, limited access to purified water [2] and extreme variations in iodine concentrations in camel milk, the association between the hydration status of camels and iodine in milk likely merits further attention.

### Iodine in water

According to WHO, there is insufficient data to derive a value for the recommended iodine content in drinking water [43]. However, several Chinese studies have found harmful health effects of median water iodine concentrations between 150–300 μg/L [44, 45], corresponding to the median level found in high water iodine areas in our study. Meanwhile, from 2013 surveys have shown reduced iodine levels in drinking water [27, 46], likely due to an increased share of purified water provided in El Aiun and Awserd [2]. The difference in excessive iodine intake from animal milk between areas with moderate and high iodine content in water as shown in this study, underlines the importance of animal drinking water as an indirect cause of iodine excess in Saharawi women, and may partly explain why the iodine status among the population remains high [20, 21]. Since 2009, Saharawi authorities have recommended that the population should use purified water both for human and animal consumption while in our study, households particularly in El Aiun and the Free Zone used wells for animal water. In the Free Zone, long distances and size of herds makes provision of purified water for animals seem highly unrealistic. Finally, provision of purified water is estimated at 15–17 L/person/day across all camps, which is comparable to what is provided to other refugee populations [2], but is most likely largely insufficient for both animal and human consumption in families owning livestock.

### Strengths and weaknesses

The main strength of our study is the large number of samples of animal milk and samples of water for both human and animal consumption collected. Together with detailed data on amounts consumed, this allowed us to assess the risk of exceeding upper intake level of iodine from an important iodine source in Saharawi women. Our study also had some weaknesses. First, for a comprehensive risk assessment, other dietary sources of iodine should have been assessed. Further, due to few samples, the study lacked power to detect causes of high iodine content in camel milk. In addition, for a more comprehensive understanding of the causes of high iodine content in animal milk in this area, data on types of feed and amounts consumed together with analysis of iodine content in animal feed would be necessary. Finally, due to lack of data on amounts of water consumed by the animals, associations between iodine in animal drinking water and iodine in animal milk should be interpreted with some caution.

## Conclusions

Animal milk is an important part of Saharawi food culture. At the same time consumption of small amounts of animal milk, in particular camel milk, may cause iodine intakes exceeding EFSAs upper intake level in Saharawi women. Iodine content in animal drinking water is likely an indirect cause of excessive iodine intake through animal milk. A further reduction of iodine in water may thus be of importance to reduce the iodine intake among Saharawi refugees. As a first step, purified drinking water should be provided continuously in all camps for both human and animal consumption.

## Supporting information

S1 Questionnaire Milk intake English. (PDF)

S2 Questionnaire Milk intake Spanish. (PDF)

S3 Questionnaire Animal feed English. (PDF)

S4 Questionnaire Animal feed Spanish. (PDF)
