# Supplementary material for: High iodine content in local animal milk and risk of exceeding EFSA upper intake level for iodine among Saharawi women
Source: PLoS One. 2019 Feb 15;14(2):e0212465. doi: 10.1371/journal.pone.0212465 (PMC6377136; doi:10.1371/journal.pone.0212465)
Supplement: S1 Questionnaire — (PDF) [file pone.0212465.s001.pdf]

Appendix 1: Questionnaire on milk consumption

Project ID number:	Camp:
Date of interview:	
Time start of interview:	Time interview finished:
Initials of field worker:	

The person answering the questionnaire is:

☐ *Mother of the youngest child in the household over 1 year*

☐ *Person responsible for the youngest child in the household over 1 year*

☐ *Youngest female of more than 16 years present in the household*

1. Do you or your family drink milk?

   ☐ Yes ☐ No

   If no: stop interview

2. If yes, what kinds of milk?

   ☐ Goat milk ☐ Camel milk ☐ Cow's milk ☐ Powder milk
   ☐ Candida milk ☐ Other, specify: _______________

Goat milk (only if answered goat milk on question 2):

3. Does the goat milk you drink come from the family's goat/s?

   ☐ Always ☐ Mostly ☐ Seldom ☐ Never

4. Did you drink goat milk, warm or cold, in the last 24 hours?

   ☐ Yes ☐ No

5. If yes, how many times?

   ☐ 1-2 times ☐ 3-4 times ☐ 5-6 times ☐ More than 6 times

6. Did you mix the goat milk with water when YOU drank it?

☐ Yes ☐ No

7. If yes, how much water in the bowl? _______ ml/_______ml (total in the bowl)
8. .How much milk (mixed with water) did you drink each time? _______ml

9. How many times did you drink goat milk in the past week? _________________

10. If respondent is mother of children or responsible for children *(If more than four, the four youngest are included. Only children under 12 years):*

	10.1<br><br>Drinks goat milk (Y/N)	10.2<br><br>Drank goat milk last 24 hours (Y/N)	10.3<br><br>How many times?	10.4<br><br>Is the milk mixed with water? (Y/N)	10.5<br><br>If yes, how much water?<br><br>(ml/ml in total)	10.6<br><br>How much milk mixed with water did the child drink each time?	10.7<br><br>How many times in the last 7 days?
Child 1, Age:							
Child 2, Age:							
Child 3, Age:							
Child 4, Age:							

Camel milk (only if answered camel milk on question 2):

11. Does the camel milk you drink come from the family's camel/s?

☐ Always ☐ Mostly ☐ Seldom ☐ Never

12. Did you drink camel milk, warm or cold, in the last 24 hours?

☐ Yes ☐ No

13. If yes, how many times?

☐ 1-2 times ☐ 3-4 times ☐ 5-6 times ☐ More than 6 times

14. Did you mix the camel milk with water when YOU drank it?

☐ Yes ☐ No

15. If yes, how much water in the bowl? _______ml/_______ml (total in bowl)

16. How much milk (and water) did you drink each time? ______ml

17. How many times did you drink camel milk in the last week? __________

18. If respondent is mother of children or responsible for children (*If more than four children, the four youngest are included. Only children under 12 years*):

	18.1 Drinks camel milk (Y/N)	18.2 Drank camel milk last 24 hours (Y/N)	18.3 How many times?	18.4 Is the milk mixed with water? (Y/N)	18.5 If yes, how much water?	18.6 How much milk mixed with water did the child drink each time?	18.7 How many times in the last 7 days?
Child 1, Age:							
Child 2, Age:							
Child 3, Age:							
Child 4, Age:							

19. Other kinds of milk the children drink? ________________________________

Drinking water:

20. From where does the family get drinking water?

☐ General water system ☐ Well/bore hole by the house ☐ Vendor
☐ Other, specify: ____________________
