# Supplementary material for: High iodine content in local animal milk and risk of exceeding EFSA upper intake level for iodine among Saharawi women
Source: PLoS One. 2019 Feb 15;14(2):e0212465. doi: 10.1371/journal.pone.0212465 (PMC6377136; doi:10.1371/journal.pone.0212465)
Supplement: S2 Questionnaire — (PDF) [file pone.0212465.s002.pdf]

Número de identificación:

Cuestionario sobre el consumo de leche

Número de identificación:	Willaya:
Fecha:	
Hora de inicio de la entrevista:	Hora final de la entrevista:
Iniciales del trabajador del proyecto:	

La persona que respondió el cuestionario es:

☐ M*adre del niño más pequeño en el hogar con más de 1 año*

☐ *Persona responsable del niño más pequeño en el hogar con más de 1 año*

☐ *Mujer más joven, mayor de 16 años, presente en el hogar*

1. ¿Usted o su familia beben leche?

   ☐ Si ☐ No

   Si no: dejar de entrevistar

2. En caso afirmativo. ¿Qué tipo de leche?

   ☐ Leche de cabra ☐ Leche de camella ☐ Leche de vaca
   ☐ Leche en polvo ☐ Leche Candia ☐ Otros, especifique: ___________

La leche de cabra (sólo si responde a la leche de cabra en la pregunta 2):

3. ¿La leche de cabra que bebe proviene de cabras de la familia?

   ☐ Siempre ☐ Parcialmente ☐ Pocas veces ☐ Nunca

4. ¿Bebió leche de cabra, caliente o frío, en las últimas 24 horas?

   ☐ Si ☐ No

5. En caso afirmativo. ¿Cuántas veces la ha tomado?

   ☐ 1-2 veces ☐ 3-4 veces ☐ 5-6 veces ☐ Más de 6 veces

6. ¿Habia usted mezclado la leche de cabra con agua, cuando se la bebió?

   ☐ Si ☐ No

7. En caso afirmativo. ¿La cantidad de agua en el recipiente? _____ml /_____ml (totales en el bol)

8. ¿Qué es la cantidad totale de leche mezclada con agua que habeis bebido cada vez.? ________ml

9. ¿Cuántas veces has bebido leche de cabra en la última semana? ____________

10. *Si la/el encuestada/o es la madre de los niños o responsable de los niños (Si son más de cuatro, los cuatro jóvenes deben incluirse. Incluir sólo los niños menores de 12 años):*

	10.1 ¿Beben leche de cabra? (S/N)	10.2 ¿Han tomado leche de cabra las últimas 24 horas? (S/N)	10.3 ¿Cuantas veces?	10.4 ¿La leche está mezclada con agua? (S/N)	10.5 En caso afirmativo, ¿Con que cantidad de agua? (ml/ml totales)	10.6 ¿Qué cantidad de leche mezclada con agua se toma cada vez ?	10.7 ¿Cuántas veces ha tomado en los ultimos 7 días?
Nino 1 Edad:							
Nino 2 Edad:							
Nino 3 Edad:							
Nino 4 Edad:							

La leche de camella (sólo si responde a la leche de camella en la pregunta 2):

11. ¿La leche de camella que bebe proviene de los camellas de la familia?

☐ Siempre ☐ Parcialmente ☐ Pocas veces ☐ Nunca

12. ¿Bebió leche de camella, caliente o fría, en las últimas 24 horas?

☐ Si ☐ No

13. En caso afirmativo. ¿Cuantas veces han sido?

☐ 1-2 véces ☐ 3-4 véces ☐ 5-6 véces ☐ Más de 6 véces

14. ¿Habia usted mezclado la leche de camella con agua cuando la bebió?

☐ Si ☐ No

15. En caso afirmativo. ¿La cantidad de agua en el recipiente? _____ml /_____ml (totales en el bol)

16. ¿Qué es la cantidad totale de leche mezclada con agua que habeis bebido cada vez.? ________ml

17. ¿Cuántas veces has bebido leche de cabra en la última semana? ____________

18. *Si el/la encuestado/a es la madre de los niños o responsable de los niños (Si son más de cuatro, los cuatro jóvenes deben incluirse. Incluir solo a los niños menores de 12 años):*

	18.1 ¿Beben leche de camella? (S/N)	18.2 ¿Han tomado leche de camella las últimas 24 horas? (S/N)	18.3 ¿Quantas veces?	18.4 ¿Está la leche mezclada con agua? (S/N)	18.5 En caso afirmativo, ¿Con que cantidad de agua? (ml/ml totales)	18.6 ¿Qué Cantidad de leche mezclada con agua se toma cada vez ?	18.7 ¿Cuántas veces ha tomado en los ultimos 7 días?
Nino 1 Edad:							
Nino 2 Edad:							
Nino 3 Edad:							
Nino 4 Edad:							

19. ¿Otros tipos de leche que tomaron los niños? _________________________

Agua potable:

20. ¿Dónde viene la agua potable que toma la familia?

☐ De sistema distribucion general ☐ De pozo familial ☐ Vendedores
☐ Agua mineral de botellas ☐ Otros, especificar: ___________________
