# Supplementary material for: High iodine content in local animal milk and risk of exceeding EFSA upper intake level for iodine among Saharawi women
Source: PLoS One. 2019 Feb 15;14(2):e0212465. doi: 10.1371/journal.pone.0212465 (PMC6377136; doi:10.1371/journal.pone.0212465)
Supplement: S3 Questionnaire — (PDF) [file pone.0212465.s003.pdf]

Appendix 2: Questionnaire on animal feeding

Project ID number:	Camp:
Date of interview:	
Time start of interview:	Time interview finished:
Initials of field worker:	

Sample collected: ☐ Goat milk (L1) ☐ Camel milk (L2) ☐ Water

(Person responsible for animals in the household):

<u>Goats:</u>

1. Does your household own goats?

   ☐ Yes ☐No

2. If yes, how many goats ("cabras")? _____ How many males? _____ How many sheep? _____

3. Do you keep your goats in a paddock?

   ☐ Yes ☐No

4. About how much milk do you get from your goats in total in the milking season per day? _______________

5. Do you or your family drink this milk?

   ☐ Yes ☐ No

6. Do you sell the milk?

   ☐ Yes ☐ No

Goat feed:

7. Does/do your goat/s ever eat household food waste?

   ☐ Yes ☐ No

8. If yes, how often?

☐ Every day ☐ 4-6 times per week ☐ 2-3 times per week
☐ Once a week ☐ 1-2 times a month

9. Does/do your goat/s ever eat carton?

☐ Yes ☐ No

10. If yes, how often?

☐ Every day ☐ 4-6 times per week ☐ 2-3 times per week
☐ Once a week ☐ 1-2 times a month

11. Does/do your goat/s ever eat waste when roaming freely?

☐ Yes ☐ No ☐ Do not know

12. If yes, how often?

☐ Every day ☐ 4-6 times per week ☐ 2-3 times per week
☐ Once a week ☐ 1-2 times a month ☐ Do not know

13. Does/do your goat/s ever eat greens (fresh grass, plants etc)

☐ Yes ☐ No

14. If yes, how often?

☐ Every day ☐ 4-6 times per week ☐ 2-3 times per week
☐ Once a week ☐ 1-2 times a month

15. Does/do your goat/s ever eat hay (dried grass)?

☐ Yes ☐ No

16. If yes, how often?

☐ Every day ☐ 4-6 times per week ☐ 2-3 times per week
☐ Once a week ☐ 1-2 times a month

17. Where does/do the goat/s get drinking water come from?

☐ Same as family ☐ External well (by paddock) ☐ Other: ______________

Camel:

18. Does your household own camels?

☐ Yes ☐No

19. If yes, how many camels? ________ How many males? _______

20. Do you keep your goats in a paddock?

☐ Yes ☐No

21. About how much milk do you get in total each day from your camels during milking season? _____________

22. Do you or your family drink this milk?

☐ Yes ☐ No

23. Do you sell the milk?

☐ Yes ☐ No

Camel feed:

24. Does/do your camel/s ever eat household food waste?

☐ Yes ☐ No

25. If yes, how often?

☐Every day ☐ 4-6 times per week ☐ 2-3 times per week
☐ Once a week ☐1-2 times a month

26. Does/do your camel/s ever eat carton?

☐ Yes ☐ No

27. If yes, how often?

☐Every day ☐ 4-6 times per week ☐ 2-3 times per week
☐ Once a week ☐1-2 times a month

28. Does/do your camel/s ever eat waste when roaming freely?

☐ Yes ☐ No ☐ Do not know

29. If yes, how often?

☐ Every day ☐ 4-6 times per week ☐ 2-3 times per week
☐ Once a week ☐ 1-2 times a month ☐ Do not know

30. Does/do your camel/s ever eat greens (fresh grass, plants etc)

☐ Yes ☐ No

31. If yes, how often?

☐ Every day ☐ 4-6 times per week ☐ 2-3 times per week
☐ Once a week ☐ 1-2 times a month

32. Does/do your camel/s ever eat hay (dried grass)?

☐ Yes ☐ No

33. If yes, how often?

☐ Every day ☐ 4-6 times per week ☐ 2-3 times per week
☐ Once a week ☐ 1-2 times a month

34. Where does/do the camel/s get drinking water come from?

☐ Same as family ☐ External well (by paddock) ☐ Other: _______________
