# Supplementary material for: High iodine content in local animal milk and risk of exceeding EFSA upper intake level for iodine among Saharawi women
Source: PLoS One. 2019 Feb 15;14(2):e0212465. doi: 10.1371/journal.pone.0212465 (PMC6377136; doi:10.1371/journal.pone.0212465)
Supplement: S4 Questionnaire — (PDF) [file pone.0212465.s004.pdf]

Numéro de identificación:

Cuestionario sobre la alimentación de los animales

Número de identificación:	Willaya:
Fecha:	
Hora de inicio de la entrevista:	Hora final de la entrevista:
Iniciales del trabajador del proyecto:	

Muestra cogida: ☐ Leche de cabra (L1) ☐ Leche de camella (L2) ☐ Agua

(La persona responsable de los animales en el hogar):

<u>Cabras:</u>

1. ¿Tienen cabras?

   ☐ Si ☐ No

   Si no, empezaîs las preguntas de camellos (página 3)

2. En caso afirmativo. ¿Cuantas cabras en totale? ______ ¿Cuantos machos?______ ¿Cuantas ovejas?______

3. ¿Tienen sus propios corrales los cabras?

   ☐ Yes ☐ No

4. ¿Cuánta leche da su/s cabra/s por día? _______________

5. ¿Usted o su familia beben esta leche?

   ☐ Si ☐ No

6. ¿Venden la leche?

   ☐ Si ☐ No

Piensos cabra:

7. ¿Comen tu/s cabra/s alguna vez los restos de alimentos del hogar?

   ☐ Si ☐ No

8. En caso afirmativo. ¿Con qué frecuencia?

☐ Cada día ☐ 4-6 veces per semana ☐ 2-3 veces per semana
☐ Una vez al semana ☐ 1-2 veces al mes

9. ¿Comen tu/s cabra/s alguna vez cartón?

☐ Si ☐ No

10. En caso afirmativo. ¿Con qué frecuencia?

☐ Cada día ☐ 4-6 veces per semana ☐ 2-3 veces per semana
☐ Una vez al semana ☐ 1-2 veces al mes

11. ¿Comen tu/s cabra/s alguna vez los residuos libremente en la naturaleza?

☐ Si ☐ No ☐ No sabe

12. En caso afirmativo. ¿Con qué frecuencia?

☐ Cada día ☐ 4-6 veces per semana ☐ 2-3 veces per semana
☐ Una vez al semana ☐ 1-2 veces al mes

13. ¿Comen tu/s cabra/s alguna vez verduras (hierba fresca, plantas, etc)

☐ Si ☐ No

14. En caso afirmativo. ¿Con qué frecuencia?

☐ Cada día ☐ 4-6 veces per semana ☐ 2-3 veces per semana
☐ Una vez al semana ☐ 1-2 veces al mes

15. ¿Comen tu/s cabra/s alguna vez heno (pasto seco)?

☐ Si ☐ No

16. En caso afirmativo. ¿Con qué frecuencia?

☐ Cada día ☐ 4-6 veces per semana ☐ 2-3 veces per semana
☐ Una vez al semana ☐ 1-2 veces al mes

17. ¿De dónde viene el agua potable para la/s cabra/s?

☐ Igual que la familia ☐ Pozo (cerca de los corrales) ☐ Otro: _______________

Camellos:

18. ¿Tienen camellos?

☐ Si ☐No

Si no: dejar de entrevistar

19. En caso afirmativo. ¿Cuantos camellos en total? _____ ¿Cuantos machos?______

20. ¿Tienen sus propios corrales los camellos?

☐ Si ☐No

21. ¿Cuánta leche da su/s camella/s por día? _______________

22. ¿Usted o su familia beben esta leche?

☐ Si ☐ No

23. ¿Venden la leche?

☐ Si ☐ No

Piensos camello/s:

24. ¿Comen tu/s camello/s alguna vez los restos de alimentos del hogar?

☐ Si ☐ No

25. En caso afirmativo. ¿Con qué frecuencia?

☐ Cada día ☐ 4-6 veces per semana ☐ 2-3 veces per semana
☐ Una vez al semana ☐ 1-2 veces al mes

26. ¿Comen tu/s camello/s alguna vez cartón?

☐ Si ☐ No

27. En caso afirmativo. ¿Con qué frecuencia?

☐ Cada día ☐ 4-6 veces per semana ☐ 2-3 veces per semana
☐ Una vez al semana ☐ 1-2 veces al mes

28. ¿Comen tu/s cameillo/s alguna vez los residuos libremente en la naturaleza?

☐ Si ☐ No ☐ No sabe

29. En caso afirmativo. ¿Con qué frecuencia?

☐ Cada día ☐ 4-6 veces per semana ☐ 2-3 veces per semana
☐ Una vez al semana ☐ 1-2 veces al mes

30. ¿Comen tu/s camello/s alguna vez verduras (hierba fresca, plantas, etc)?

☐ Si ☐ No

31. En caso afirmativo. ¿Con qué frecuencia?

☐ Cada día ☐ 4-6 veces per semana ☐ 2-3 veces per semana
☐ Una vez al semana ☐ 1-2 veces al mes

32. ¿Comen tu/s camello/s alguna vez heno (pasto seco)?

☐ Si ☐ No

33. En caso afirmativo. ¿Con qué frecuencia?

☐ Cada día ☐ 4-6 veces per semana ☐ 2-3 veces per semana
☐ Una vez al semana ☐ 1-2 veces al mes

34. ¿De dónde viene el agua potable para las cameillos?

☐ Igual que la familia ☐ Pozo (cerca de los corrales) ☐ Otro: _______________
